# Pharmaceutical systems strengthening through the lens of Tony Boni – a tribute

**DOI:** 10.1080/20523211.2024.2305775

**Published:** 2024-01-25

**Authors:** Jude Nwokike, Maria Miralles, Douglas Keene, Patrick Lukulay, Alison Collins, Elizabeth Ludeman, Tobey Busch, Francis Aboagye-Nyame, David Lee Chin

**Affiliations:** a United States Pharmacopeia, Rockville, MD, USA; b Formerly with United States Agency for International Development, Washington, DC, USA; c Formerly with Management Sciences for Health, Arlington, VA, USA; d Technology Solutions for Global Health, Accra, Ghana; e United States Agency for International Development, Washington, DC, USA; f Management Sciences for Health, Arlington, VA, USA

## Abstract

With clarity of vision and ability to navigate complex government bureaucracy, Anthony (Tony) Boni built an enduring legacy in pharmaceutical systems strengthening.

Very few experts could muster the clarity of his vision, and yet he only stumbled into the profession. Without formal training in public health or related discipline, Anthony (Tony) Boni played a larger-than-life role in steering the emergence of transformative development assistance for improving access to life-saving pharmaceutical products and services. Starting with work in contraceptives and family planning he grew in his understanding of the need for advocacy for pharmaceuticals. The support for pharmaceutical systems strengthening, as he initially conceptualised, was intended to be implemented by two technical partners, one for country-level supply management technical assistance provision and the other for medicines information and product quality control and quality assurance. The vision evolved concomitantly, as experts helped shape his thinking in the complex history of three decades of pharmaceutical system strengthening. Populations in countries in nearly 50 countries around the world have been touched through his advocacy and the various USAID-funded programmes that he designed. His legacy will live on through the hundreds of young professionals he recruited as progenies and partners from these countries in the shared agenda to improve access to and appropriate use of quality-assured essential medicines. This approach later proved to be an effective doctrine for building capacity at the country level that was effective and sustainable. He had the unique ability to identify talents in people and nurture them. Today, countless global leaders are testament to Tony’s unique ability.

Tony was masterful in his ability to navigate complex government bureaucracy and apply his entrepreneurial talents. His effective advocacy helped to develop and oversee the implementation of multiple global US government-funded programmes. An early champion for expanding access to medicines, Tony appreciated that drug donation programmes serve as a temporary balm. He advocated for the development of capacity in low- and middle-income countries (LMICs) to manage pharmaceutical procurement and ensure the availability of essential medicines. The ‘availability’ focus soon transitioned to ‘access’, in which the access framework included dimensions of availability, affordability, accessibility, and acceptability. Tony helped spearhead this global discussion on the domains of access with MSH, Harvard, and WHO (Center for Pharmaceutical Management, 2000). And by the time that became an accepted mission, Tony had already envisioned the next mission – that access to medical products alone is insufficient. Appropriate use and delivery of pharmaceutical services are equally important for optimal health outcomes. Always a great communicator with a keen interest to get his message across, he tagged onto an accepted slogan to simplify his message; Tony adopted the slogan ‘No product No program’ slogan and added, ‘no services no health outcomes’. Applying that to emerging global health problems, he advocated for pharmaceutical system professionals to be included in the first HIV/AIDS operations research study to determine if and how AIDS medicines could be delivered and managed, and services provided in developing countries.

In 2017, the United States Pharmacopeia, (USP) held an event to celebrate 25 years of collaboration between USP and USAID. Featuring Tony Boni, who had as of then retired from USAID, the event aimed to capture early thinking in the development of programmes from 1992 to 2016 related to quality assurance systems. That event provided an opportunity to chronicle Tony’s career, his contributions, and his insights into the past and future of pharmaceutical systems strengthening. He was endlessly captivated to discuss the role of a pharmaceutical subsystem of the health system, measures of performance and sustainability of pharmaceutical systems, the difference between quality control and quality assurance, the relationship between poor quality antibiotics and antimicrobial resistance, the differentiation of long-term systems strengthening strategies from immediate system support interventions, etc. According to Tony, the

advantage of taking a systems strengthening perspective is that it allows for thinking through all of the moving parts, that is, all of the health system building blocks in a nonlinear manner. In this way, we can uncover the inter-relationships between the building blocks and critically assess the implications of corrective actions for the health system in the short, medium, and long terms, and not just for the immediate functional area of interest.

On the burden of substandard and falsified medicines, Tony postulated that poor-quality medical products not only represent a serious public health problem but also threaten to undermine the huge investments made by global health initiatives in expanding access to treatment for major diseases. He had an unquenchable appetite to understand the work of national quality control laboratories and how the results they generate can support regulatory actions. At his motivation, the Drug Quality and Information (DQI) programme that was funded by USAID and implemented by USP developed the guidelines for Ensuring the Quality of Medicines in Resource-Limited Countries (United States Pharmacopeia, 2007) in collaboration with the WHO, National Drug Authority of Uganda, Program for Appropriate Technology in Health, National Pharmaceutical Control Bureau of Malaysia, National Institute for Drug Quality Control of Vietnam, Medicines Control Authority of Zimbabwe, Organon International, and the USAID-funded Rational Pharmaceutical Management Plus Program implemented by Management Sciences for Health. Published in 2007, the operational guide quickly became a resource for the assessments of LMICs quality assurance systems. The presence of substandard and falsified medicines is but one performance indicator of a regulatory system failure, not just the failure of one regulatory function. Countries are confronted with systemic challenges that can be measured in terms of backlogs of drug registration applications awaiting review, regulatory processes that are not conducted in a transparent and accountable manner, and inadequate reporting systems to monitor therapeutic effectiveness, among others. After the WHO medicines prequalification programme was launched in 2001 to support procurement by UN agencies (‘t Hoen et al., 2014), Tony immediately recognised the need for the provision of technical assistance to emerging generic drug manufacturers to enable them to meet good manufacturing practices requirements of the WHO prequalification programme. Technical assistance deployed through USAID’s Promoting the Quality of Medicines (PQM) programme and its successor, the PQM+ programme, has contributed significantly to increasing the number of prequalified products for global health programmes. Tony had argued that without quality-assured medicines, all other health investments are negated.

One example of a project that demonstrated Tony’s entrepreneurial spirit was the collaboration with the US FDA to establish an interagency agreement to study pharmacovigilance systems in Africa and Asia. For two years, USAID directed FDA funding to apply technical approaches from the Supporting Pharmacovigilance in Developing Countries: The Systems Perspective (Strengthening Pharmaceutical Systems (SPS), 2009) and Indicator-based Pharmacovigilance Assessment Tool (IPAT) (Strengthening Pharmaceutical Systems (SPS) Program, 2009) to measure the maturity of pharmacovigilance systems in those two regions of the world. Findings from that work have informed the development of passive and active pharmacovigilance systems in more than 40 countries to support the deployment of new medicines for HIV/AIDS, Tb, and malaria. Tony had tremendous respect for experts and could immediately recognise valuable technical approaches that deliver impact. He recognised the value of active safety surveillance programmes to support the deployment of new medicines. Active surveillance provides denominator data to determine the incidence of adverse reactions and the risk factors associated with them. These are critical information that helps for a better understanding of the safety, tolerability, and effectiveness of medicines used in public health programmes and for the revision of treatment guidelines. Countries with limited pharmacovigilance capacity had over-relied on passive reporting. Tony recognised that the pharmacovigilance systems of those countries suffer from underreporting and are primarily skewed towards reporting only major events that lead to immediate harm and hospitalisation. The development of robust capacity for safety surveillance eventually surfaced as a major issue during COVID-19 when limitations in the data generated during pre-approval review and gaps in knowledge due to a lack of completeness of safety and quality data emphasised the need for strong post-marketing surveillance systems.

Throughout his career, Tony engaged with the WHO in multiple subjects that covered the development of the access framework, essential medicines list, rational drug use, adherence to antiretroviral drugs, antimicrobial resistance, regulatory systems strengthening, prequalification programme, adoption of GMP standards, etc. His contributions to several global initiatives in collaboration with other global players are too numerous to mention. One that merits special mention is the International Network for Rational Use of Drugs (INRUD) (Ross-Degnan et al., 1992), established in 1989 to promote the safe, effective, and cost-effective use of medicines. The network met every 7 years to discuss progress in the design and implementation of strategies to improve rational use of medicines. INRUD received support for its first 10 years mainly from the Danish Agency for International Development (Danida). Tony advocated for USAID support to the INRUD secretariat in the late 2000s once the network was functioning, including support to several INRUD country-specific operational research activities. USAID was involved in the Joint Initiative for Research on Improving Use of Medicines (JIRIUM), following the first International Conference on Improving Use of Medicines in Chiang Mai, Thailand (1997).

In retirement, Tony remained engaged in developments in pharmaceutical systems strengthening. Tony was interested in documenting the evolution of issues in pharmaceutical management. He concluded that the value of quality assurance and regulatory systems strengthening in development assistance is now well recognised. However, the issue of substandard and falsified medicines is not completely resolved. Not until the universal adoption of GMP standards by manufacturers, risk-based deployment of limited regulatory resources, adoption of regulatory collaboration, reliance, and work-sharing for sustainable surveillance activities that guarantee the quality and integrity of the products throughout the supply chain.

Friends, colleagues, and mentees also knew Tony as a talented amateur magician. He offered magic as an icebreaker into serious discussions. In a sense, the readiness to open his magic box and play at the slightest request was not only a prelude to serious pharmaceutical management talks but allegorically like his passion for helping people in any way he could. A shy yet generous person at heart, magic helped Tony meet people and nurture long-lasting relationships with them.

In conclusion, this tribute serves as a testament to the unwavering dedication and profound impact of an exceptional advocate and mentor in the field of pharmaceutical system strengthening. Through his unparalleled commitment, visionary leadership, and unyielding passion, he has illuminated a path towards more robust and resilient pharmaceutical systems in low- and middle-income countries. Tony’s remarkable ability to inspire, influence, and drive change will continue to reverberate through the endeavours of those fortunate enough to have learned from his wisdom.

As we honour his remarkable contributions, let us reflect on the profound difference they have made to health systems and recommit ourselves to the ideals they championed. Through his indomitable spirit and tireless efforts, he has instilled a sense of purpose and determination in us all, leaving an indelible mark on the landscape of pharmaceutical system strengthening that will be felt for years to come.
